# Global Gradients in Vertebrate Diversity Predicted by Historical Area-Productivity Dynamics and Contemporary Environment

**DOI:** 10.1371/journal.pbio.1001292

**Published:** 2012-03-27

**Authors:** Walter Jetz, Paul V. A. Fine

**Affiliations:** 1 Department of Ecology and Evolutionary Biology, Yale University, New Haven, Connecticut, United States of America; 2 Department of Integrative Biology, University of California, Berkeley, California, United States of America; Imperial College London, United Kingdom

## Abstract

Broad-scale geographic gradients in species richness have now been extensively documented, but their historical underpinning is still not well understood. While the importance of productivity, temperature, and a scale dependence of the determinants of diversity is broadly acknowledged, we argue here that limitation to a single analysis scale and data pseudo-replication have impeded an integrated evolutionary and ecological understanding of diversity gradients. We develop and apply a hierarchical analysis framework for global diversity gradients that incorporates an explicit accounting of past environmental variation and provides an appropriate measurement of richness. Due to environmental niche conservatism, organisms generally reside in climatically defined bioregions, or “evolutionary arenas,” characterized by in situ speciation and extinction. These bioregions differ in age and their total productivity and have varied over time in area and energy available for diversification. We show that, consistently across the four major terrestrial vertebrate groups, current-day species richness of the world's main 32 bioregions is best explained by a model that integrates area and productivity over geological time together with temperature. Adding finer scale variation in energy availability as an ecological predictor of within-bioregional patterns of richness explains much of the remaining global variation in richness at the 110 km grain. These results highlight the separate evolutionary and ecological effects of energy availability and provide a first conceptual and empirical integration of the key drivers of broad-scale richness gradients. Avoiding the pseudo-replication that hampers the evolutionary interpretation of non-hierarchical macroecological analyses, our findings integrate evolutionary and ecological mechanisms at their most relevant scales and offer a new synthesis regarding global diversity gradients.

## Abstract 2

A novel hierarchical framework integrates the effects of time, area, productivity, and temperature at their respective relevant scales and successfully predicts the latitudinal gradient in global vertebrate diversity.

## Author Summary

Understanding what determines the distribution of biodiversity across the planet remains one of the critical challenges in biology and has gained particular urgency in the face of environmental change and accelerating species extinctions. Our study develops a novel analytical framework to jointly evaluate historical and contemporary environmental predictors of the latitudinal gradient in the diversity of terrestrial vertebrates. The number of vertebrate species is greater in warm, productive biomes, such as tropical forests, that have both a large size and a long evolutionary history. Using just a few key predictor variables—time, area, productivity, and temperature—we are now able to explain more than 80% of the variability in biodiversity among bioregions. By integrating each of these factors at both the regional and local scale in a hierarchical model, we are able to provide a consensus explanation for broad-scale diversity gradients that encompasses both ecological and evolutionary mechanisms.

## Introduction

The uneven distribution of species diversity is a key feature of life on Earth and has myriad implications. While the scale-dependence of the determinants of the global variation in diversity is well acknowledged [1]–[6], to date a quantitative accounting of the roles of history and environment in generating and maintaining gradients in species richness is still lacking. Over the past three decades, increased data availability has facilitated analyses of contiguous geographic patterns in species richness at relatively fine spatial grains (100–200 km) at both continental [7]–[9] and global scales [10],[11]. At these spatial resolutions, environmental variables such as productivity or temperature have been shown to offer extremely strong statistical predictions of species richness [8],[11]–[18]. However, it has been difficult to connect these results directly with underlying evolutionary and ecological processes. One problem is that the ultimate drivers underpinning diversity, namely speciation and extinction [19], operate at scales much larger than the spatial resolution (e.g., 100 km grids) of most analyses. A number of studies have confirmed the strong effect of regional richness on local richness [1]–[3],[6],[20] and have speculated on the role of energy driving diversification at regional scales [21]–[24] as well as sorting at local scales [25]–[27]. But attempts to integrate them at the appropriate scale have been limited, and we know of no study that has quantified the effect of productivity on richness gradients jointly at regional and local scales and both in terms of evolutionary and ecological processes.

Another impediment to interpretations of gridded richness analyses has been that species' geographic ranges are generally much larger than, for example, 100 km×100 km grid cells, resulting in geographically non-random patterns of pseudo-replication, inflated spatial autocorrelation, and an overrepresentation of wide-ranging species and their respective climatic associations [8],[28]. These issues have to date precluded straightforward evolutionary and ecological interpretations of macroecological environment correlations of gridded richness patterns [5],[29]. While partly motivated by limits in the knowledge of fine-scale species distributions [30], macroecological analyses have also been conducted using, for example, ca. 800 ecoregions as spatial units [14],[31], but these regions still incur significant and geographically variable redundancy in species. We are not aware of a study on richness gradients that has successfully overcome this problem and thus truly have given each species equal weight.

Finally, while there is little doubt about the importance of time for diversification [32]–[34], attempts to date to invoke paleoclimate for understanding richness have been hampered by the lack of data, especially at deeper time scales. Several studies have linked relatively recent climatic oscillations, for example, those causing quarternary high-latitude glaciation, to geographic richness patterns [22],[35]–[37]. The geography of deeper time climate conditions and exactly how it relates to the tempo of past clade diversification is inherently difficult to estimate. But given deep conservatism in the environmental (e.g., biome) associations within clades [38],[39] compared to relatively dynamic geographic ranges [40], clades are expected to much more strongly track climatically defined regions, or biomes, rather than specific geographical locations over evolutionary history. The ages of biomes may thus offer a promising avenue for understanding the role of paleoclimate contributing to contemporary patterns of species richness and have recently been successfully correlated with both turtle and tree richness at the regional scale [41],[42]. To date, analyses connecting the age and area of regions to finer grain richness patterns have not been attempted.

Here, we aim to address these problems with a hierarchical framework that integrates the drivers of regional diversification of species with those of their sorting into finer grain assemblages at their respective scales of influence. We use this model to test the relative importance of past spatio-temporal variation of climatic conditions (specifically time-integrated area and productivity) versus contemporary environment for explaining both the regional and finer scale variation in the species richness of terrestrial vertebrates worldwide. Due to environmental niche conservatism, organisms are generally restricted to climatically defined bioregions, or “evolutionary arenas,” characterized by in situ speciation and extinction. We expect differences in species richness between such regions to arise from different levels of net diversification (speciation – extinction over time). The number of speciation and extinction events should vary among regions due to differences in the sizes of populations over time and the opportunities for reproductive isolation for all resident taxa [32]. We expect these drivers to be associated with today's area [29],[32],[43] and energy availability (i.e., productivity) [8],[11]–[14] of bioregions but, critically, also with the past levels of these factors—that is, how bioregions have varied in areal extent and productivity over time [42]. Furthermore, regional rates of diversification have been hypothesized to vary with temperature and its effects on activity and biological rates such as rates of molecular evolution or species interactions [15]–[17],[44]. We expect all of these drivers in concert to shape broad-scale gradients of diversity and predict that in an integrative assessment of regional differences in diversity, (i) models accounting for the temporal availability of area or productivity will outperform those without (i.e., regions that are older and/or have in the past been larger in extent will support higher vertebrate species richness than younger and/or smaller regions), (ii) area times energy availability (net primary productivity) will be a stronger predictor of richness than area alone [13],[45], and (iii) average bioregion temperature will positively affect richness above and beyond the effects of productivity and have a stronger effect in ectotherms compared to endotherms [15].

**Figure 1:**
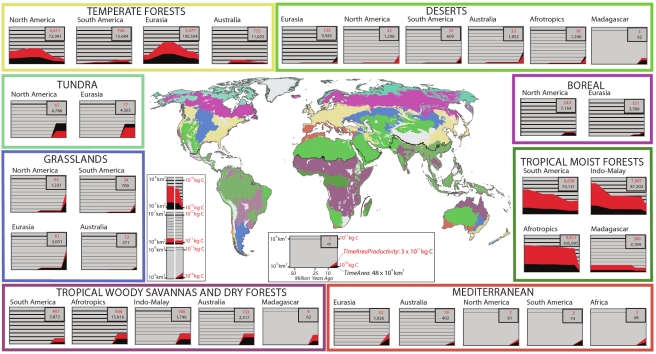
Map of study bioregions and their area and annual productivity dynamics. The variation in area (black) and annual productivity (red) over the last 55 million years forms the species richness predictors *TimeArea* (cumulative time-area, units 10^4^ km^2^×million years) and *TimeAreaProductivity* (cumulative total productivity, units 10^17^ kg Carbon), respectively (values in upper right box corner). Panel boxes have one of three different *y*-axis scales (note different line thicknesses and legend). For example, in tropical woody savannas and dry forests, the land area for the last few million years has been ∼1×10^7^ km^2^ in the Afrotropics, ∼2×10^6^ km^2^ in Australia, and ∼1×10^5^ km^2^ in Madagascar. See also Tables S1, S2, and S5.

We test these predictions for the 32 main subdivisions, or “bioregions,” of the world based on vegetation type and major landmass (Figure 1, Tables S1 and S2) [46],[47]. We excluded montane regions (which exclusively harbor ca. 5% of vertebrate species and represent ca. 15% of global land area) due to their extremely steep environmental gradients and associated species turnover, which impedes reliable bioregional delineation and estimates of their extent over time. Over historical time-scales these climatically and geographically distinct bioregions have been characterized by similar environmental and climatic conditions, but have changed in size and shape over time within their respective realms [42]. All bioregions are within the range of scales over which allopatric speciation of terrestrial vertebrate speciation typically occurs (100–1,000 km scale [48]) and may thus be considered bio-climatically and geographically distinct “evolutionary arenas.” After deriving time-integrated models of bioregion species richness, we then in a second step assess their ability to predict the variation in richness at the scale of 110 km grid cell assemblages. We make these finer scale predictions first under a model of simple random sorting of species from those predicted for the bioregion and, second, under a model of sorting mediated by the relative productivity of a grid cell. The goals of this second step include (i) an evaluation of the ability of this hierarchical model to make strong fine-scale richness predictions (while including paleoclimate and avoiding regional-level conflation of sample size) and (ii) a demonstration of the separate roles energy availability has at different temporal and spatial scales.

## Results and Discussion

### Bioregion Historical Dynamics

Paleoclimatic data reveal dramatic variation in the age and spatial dynamics of different bioregions from the end of the Paleocene (55 MY bp) to the present day (Figure 1, Table S5). For example, grasslands are not thought to have covered large areas on earth until 8 million years ago, resulting in a much smaller area over time than observed for, for example, temperate or tropical moist forests that have a longer history (Figure 1). Linking estimates of the extents of bioregions over time allows the calculation of “time-integrated area” (*TimeArea*) [42], a synthetic index of area available to the bioregion's biota over time, varying from just 48×10^4^ km^2^ integrated over 55 million years in the case of the Mediterranean bioregion at the southern tip of Africa to over 100,000×10^4^ km^2^ in Eurasian temperate and African moist tropical forests. Unlike bioregion extent and position, climatic conditions of bioregions are assumed to be relatively static over time [49], which allows the determination of average bioregion net primary productivity (*Productivity*) and *Temperature*. Summed *Productivity* over bioregion *Area* yields total bioregion productivity (*AreaProductivity*)—that is, total annual carbon flux measured in kg/year over a whole bioregion, a measure that exhibits joint dynamics with bioregion *Area*. But integrated over time in the form of *TimeAreaProductivity*, it exhibits very different geographic patterns than *TimeArea* (Figure 1) with, for example, African and IndoMalay tropical moist forests experiencing a flux of over 8,000×10^17^ kg of carbon over the past 55 million years and the Mediterranean regions of the New World and Africa just under 3×10^17^ kg.

### Bioregion Biotic Independence

We summarized terrestrial vertebrate richness per bioregion as *Total* (every species found in a bioregion), *Resident* (species for which a given bioregion contains the largest portion of the range), and *Endemic* (species that are restricted to a single bioregion; Table S4). We find minimal overlap in *Total* species among bioregions (median Jaccard similarity among bioregions: 4% for birds, 0% for other taxa; Figure S1, Table S3), which confirms their relative evolutionary isolation in addition to climatic and spatial independence and a consistently strong pattern of biome conservatism [38],[50],[51]. It also confirms that across all four vertebrate groups these selected bioregions represent useful spatial units that avoid the pseudo-replication of species: for *Resident* species richness every species enters a given analysis exactly once, and the number of distribution records is equal to the global richness of species (13,860 endothermic mammals and birds, 11,836 ectothermic amphibians and reptiles; montane endemics excluded). For *Endemic* species (total of 13,111 species and records) bioregions are even more likely to represent the true regions of origin compared to *Resident* species. We therefore expect a stronger correlation of area and productivity integrated over time (*TimeAreaProductivity*) with the diversity of *Endemic* species.

### Bioregion Species Richness

**Figure 2:**
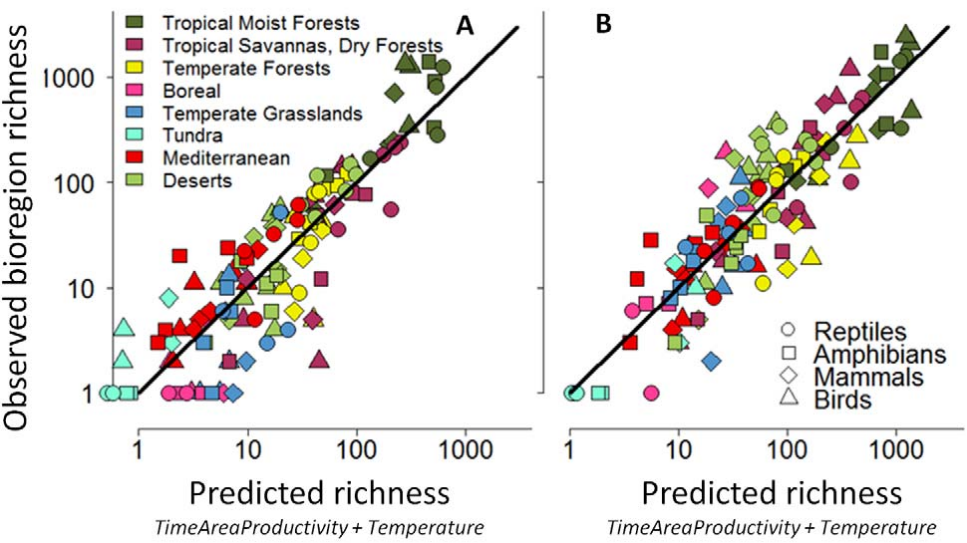
Observed versus predicted bioregion species richness of terrestrial vertebrates. Observed bioregion species richness (A, *Endemic* species, B, *Resident* species) is plotted against that predicted by the two-predictor *TimeAreaProductivity+Temperature* model fit separately for each of the four taxa (different symbols). Lines indicate least squares fit of regressions relating to observed predicted richness for each of the four taxa over the 32 bioregions (*r* ^2^ [*Endemic*] = 0.78, *r* ^2^ [*Resident*] = 0.78, *N* = 128). For detailed results, see Table S7. Colors indicate biome membership (see the map in Figure 1 to match colors).

All three predictions of our integrative model regarding the effect of time-area-productivity on richness are confirmed (Table 1). The models that account for time-integrated productivity and also include temperature as an additional predictor yield the strongest fits. For endotherms, the time-integrated measures of area outperform models that ignore time only for the *Endemic richness* dataset, which offered the more direct test of our hypotheses. Predictions of the two-predictor *TimeAreaProductivity*+*Temperature* model are consistently strong across all four vertebrate taxa, which represent independent replicates (Figure 2), explaining over 77% of the variation in richness (Figure 2, *N* = 128, see Tables S7 and S8 for more details). Models that fit *TimeArea* and *Productivity* as statistically separate terms do not on the whole yield stronger predictions (Table S9). This lends support to Wright's [45] parallel findings for large islands, which represent similarly closed systems, and contrasts with previous results reported for 110×110 km grid cells [13]. The shape of the *Productivity*-richness relationship is linear (in Endotherm *Residents*) or positive accelerating in linear space (in Ectotherm *Residents* and both *Endemics* groups). In contrast, the slopes of the *AreaProductivity-* and *TimeAreaProductivity-*richness relationships, whether fitted with or without *Temperature*, are all positive saturating—that is, species richness tends to increase more steeply in the low than in the high productivity ranges (coefficients in ln-ln space vary between 0.4 and 1, Table S7). We did not find evidence of a hump-shaped pattern for any measure of productivity and richness at the bioregional scale [52],[53].

**Table 1:** Relative performance of integrated single- and two-predictor models of bioregion species richness.

Predictor Variables	*Endemic*				*Resident*			
	Endotherms		Ectotherms		Endotherms		Ectotherms	
	Δ*AIC*	*r^2^*	Δ*AIC*	*r^2^*	Δ*AIC*	*r^2^*	Δ*AIC*	*r^2^*
*TimeAreaProductivity+Temperature*	**0**	**0.67**	**0**	**0.82**	20	0.70	**1**	**0.86**
*TimeArea+Temperature*	**0**	**0.68**	**0**	**0.82**	14	0.75	**0**	**0.87**
*AreaProductivity+Temperature*	10	0.56	13	0.73	**0**	**0.84**	3	0.85
*TimeAreaProductivity*	20	0.36	46	0.22	31	0.56	55	0.20
*TimeArea*	23	0.29	49	0.14	32	0.54	58	0.13
*AreaProductivity*	26	0.22	50	0.10	22	0.66	57	0.16
*Productivity*	26	0.23	42	0.29	52	0.12	52	0.29
*Area*	31	0.09	53	0.02	35	0.49	61	0.04
*Temperature*	22	0.31	24	0.60	52	0.14	29	0.65

*Endemic* species are those restricted to a single bioregion and *Resident* counts species with the largest portion of their range in a given bioregion. Endotherms combine mammals and birds, ectotherms combine reptiles and amphibians. Best models (ΔAIC<2) are highlighted in bold, and *r* ^2^ refers to pseudo-*r* ^2^ values based on fitting model-predicted versus observed. Note that the results for both richness values are unaffected by the pseudo-replication that hampers the results of typical gridded analyses of species richness. Predictor variables: *Temperature*, average temperature of bioregion; *Area*, current-day extent of a bioregion; *Productivity*, average bioregion productivity; *AreaProductivity*, total bioregion productivity, that is, the product of *Productivity* and bioregion *Area*. *TimeArea*, time-integrated area, that is, the integrated areal extent of a bioregion over 55 million years; *TimeAreaProductivity*, time-integrated productivity, that is, the product of *Productivity* and *TimeArea*. For further details and results by taxon, see Methods and Tables S1, S2, S3, S4, S6, S7, S8, S9, S10.

As predicted, ectotherm richness increases much more steeply and strongly with temperature than endotherm richness, both when fitted singly and when controlled for *TimeAreaProductivity* (Figures S2 and S3, Table S7). This supports at the global scale the significant and complementary effect temperature may contribute to levels of regional ectotherm diversity (see also [4],[14],[44]). For ectotherms, higher temperatures in tropical regions may be promoting higher rates of genetic incompatibilities among populations or faster rates of biotic interactions, further accelerating speciation rates [44],[54],[55]. Alternatively, the thermal dependence of activity represents a strong constraint on ectotherm distribution [56], likely imposing limits on clade origination and diversification in high-latitude regions. Third, in warm regions, ectotherms are released from physiological and behavioral adaptations to cold stress promoting a greater diversity of life histories and metabolic “niches” [57],[58]. These factors are not mutually exclusive, and more work is needed for understanding the potential role of temperature and thermal physiology in driving diversification. Preliminary results from phylogenetic analyses suggest increased diversification rates at lower latitudes in both amphibians [59] and mammals [60], but with a much weaker and more equivocal trend in the latter.

Overall, our bioregion results support the hypothesized interactions of environmental conditions and area over time in influencing the speciation and extinction and ultimately species richness of biota in bioregions. We suggest that the bioregional variation in time-integrated productivity successfully captures key factors affecting both cumulative population sizes over time as well as the different opportunities for reproductive isolation. Large, productive areas like the Neotropical moist/wet forest biome have been characterized by high productivity and a continuously large extent, and thus have supported large populations of each of the four vertebrate clades, since before the Eocene (700×10^12^ km^2^ years and 663×10^18^ kg Carbon produced since 55 MY bp; Figure 1). Reproductive isolation has been facilitated by the large amount of time that vertebrate populations have had to encounter geographical barriers (such as rivers in non-volant mammals [61]) as well as heightened habitat heterogeneity related to the high productivity (i.e., multiple vertical forest strata) [12]. This contrasts with, for example, unproductive North American deserts, which have only come to cover a substantial area within the last few million years (12×10^12^ km years and 3×10^18^ kg Carbon; Figure 1) [62]. We suggest that the large *TimeAreaProductivity* seen in, for example, the Neotropical forest compared to the North American desert bioregion in Figure 1 reflects all factors affecting cumulative population sizes over time (which have affected both speciation and extinction probabilities) as well as opportunities for reproductive isolation. Together, these factors have led to the wide discrepancy in vertebrate diversity between these two bioregions.

Previous studies have employed phylogenies or sister-group comparisons to test whether the latitudinal diversity gradient derives from more evolutionary time [63], niche conservatism [38], or differences in speciation or extinction rates at different latitudes [22],[59],[60],[64]. Factors such as orbital forcing causing glaciation at high latitudes have been posited to elevate extinction rates and are expected to accentuate the observed disparities in species richness among bioregions, especially for endemics [35],[65]. The results reported here complement these studies and suggest that at the bioregion scale, and over an extremely large window of time (55 MY), diversification rates consistently vary with respect to the area, age, and productivity of a given bioregion (Figure 2). We thus view the time-integrated productivity of bioregions to be a general explanation for why so many clades originate at lower latitudes and correspondingly fewer have diversified into bioregions at higher latitudes. It is important to note that time alone is not sufficient to explain these patterns: temperate bioregions are just as ancient as tropical bioregions but strongly differ in their cumulative time-integrated area and productivity. In sum, the strong associations we find indicate a pathway toward first-order approximations of rates of net species production per bioregion, based on variation in area over time, productivity, and temperature. Future studies could integrate our approach with more detailed comparisons of clade-level diversification rates among bioregions or combine it with existing phylogenetic methods for quantifying correlates of diversification.

### Finer Scale Species Richness

**Figure 3:**
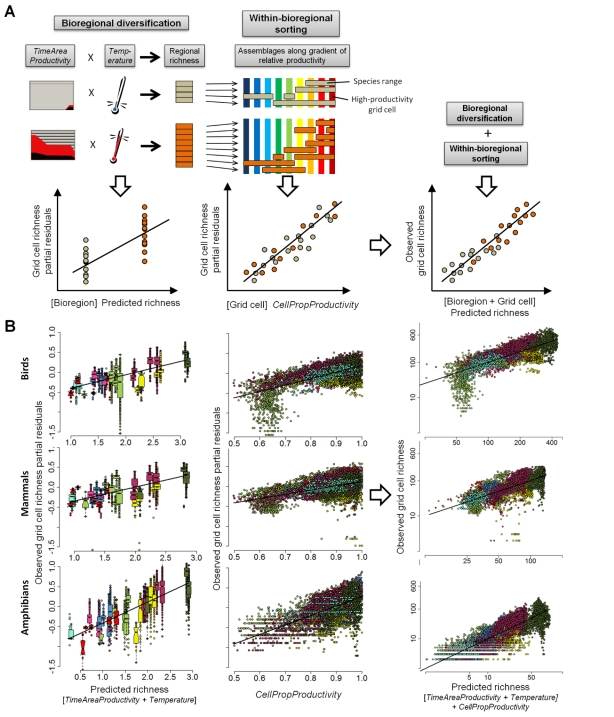
Hierarchical prediction of species richness at the scale of 110 km grid cells (*N* = 9,253). (A) Conceptual outline of the model and (B) empirical evaluation for the 110 km grid cell *Total Richness* of Mammals, Birds, and Amphibians. The model first fits differences in grid cell richness among bioregions based on the *Resident* richness model of bioregion-level diversification (*TimeAreaProductivity*, *Temperature*, see Table 1, Figure 2; additional effect of *Area* was also fitted and significant for Amphibians, see Figure S4, Table S12). Second, the effect of within-bioregion gradients in productivity (*CellPropProductivity*, i.e., proportion of bioregion grid cell maximum, a measure that standardizes productivity across bioregions) is fitted to predict subsequent sorting of each bioregions' species into grid cell assemblages. The resulting hierarchical prediction of grid cell richness accounts for the scale dependence of different effects and in the case of productivity addresses the different mechanisms of the same variable at different scales. In (B), lines indicate least squares model fits (*r* ^2^ values for observed–predicted; bioregion level, grid cell level, respectively: *r* ^2^ [Birds] = 0.40, 0.61; *r* ^2^ [Mammals] = 0.45, 0.58; *r* ^2^ [Amphibians] = 0.59, 0.77). Boxplots (left panels) summarize points for each of the 32 bioregions. Colors indicate biome membership (see Figure 2 for legend). See also Figure S4 and Tables S12 and S13. Partial residuals illustrate the relationship between a predictor and the response given other predictors in the model.

Having addressed key evolutionary drivers affecting the broad-scale variation in vertebrate diversity, we next assess how each bioregion's species sort into grid cell assemblages and how both processes combine to explain the finer scale geographic variation in richness (Figure 3A). We perform this assessment for the 18,467 bird, mammal, and amphibian species in the bioregion analysis and their 2,966,137 occurrences across the 9,253 110×110 km terrestrial grid cells encompassed by the bioregions (Figure 3B). Strong effects of regional- on fine-scale richness have previously been demonstrated [1],[2], and here we provide a first test of their pervasiveness at a global scale by evaluating the performance of bioregion models for explaining grid cell richness. We find that the two-predictor *TimeAreaProductivity+Temperature* model developed above (Table 1) alone explains 46%–60% and 32%–50% of the variation in *Resident* richness and *Total* richness, respectively (Figure 3B left column, Tables S11 and S12). This highlights how regional effects together with even simple null models of proportional sorting are able to explain much of the finer scale richness patterns. Fine-scale–regional richness relationships are known to be affected by spatial scale as well as by species' dispersal abilities [66]. In larger regions a grid cell of the same size represents a smaller portion of the regional area and, assuming similar levels of grid cell immigration/extinction, grid cell richness is expected to be smaller. This should apply whenever average species range sizes increase less than proportionally with bioregion size and should be particularly noticeable for taxa with relatively low dispersal rates or small within-bioregion range sizes (such as amphibians compared to birds or mammals), because with increasing bioregion size species will be progressively less likely to occupy a given grid cell. We find these expectations confirmed. Bioregion *Area* exhibits an additional negative effect and improves fine-scale predictions, especially for *Total* richness. It does so most strongly in Amphibians (Figure S4, Table S12), whose greater dispersal limitation (and on average by a factor of four smaller geographic ranges) compared to mammals or birds has been previously suggested as contributing to their strong patterns of species turnover [67].

Species vary strongly in the number of assemblages they occupy and the species richness of grid cell assemblages is a function of the drivers that affect species' sorting and resulting overlap in geographic ranges. One variable strongly associated with the sorting into assemblages, particularly by wide-ranging species, is local energy availability [8],[25]. We find that relative productivity in a grid cell (*CellPropProductivity*, i.e., the proportion of the maximum grid cell productivity observed in a bioregion) predicts a substantial additional amount of observed variation in grid cell richness (Figure 3B middle column, and S13) and confirms the expected greater tendency of species within a bioregion to occupy high-productivity grid cells. Allowing the shape of the richness–productivity relationship to vary among regions improves predictions (Tables S12 and S13), but only slightly so, suggesting a within-regional role of productivity that is globally fairly consistent. Nevertheless, the total amount of variation explained by the *TimeAreaProductivity*+*Temperature* model (58%–77%) is remarkable and similar to that found in previously published broad-scale gridded richness regression analyses [8],[11]. Notably, however, the hierarchical approach avoids the dual problems of species pseudo-replication and conflation of among- and within-regional processes—issues that have seriously impeded interpretations of all previous gridded biogeographic or macroecological analyses at broad scales.

Our results largely corroborate past studies that have hypothesized that net primary productivity should be a dominant predictor of fine-grain assemblage richness [8],[11],[16]. However, our hierarchical model is able to separate how productivity influences species richness at different temporal and spatial scales. At the bioregional scale, productivity should increase the cumulative population size and opportunities for reproductive isolation over time, promoting higher species richness in high-productivity bioregions [12]. At the fine scale productivity affects the occupancy of assemblages in relation to the regional pool [27],[68]. In addition to the sampling effects inherent with larger assemblage-level population sizes, increased productivity may promote greater richness due to an increased number of niches facilitating species coexistence [12],[25].

### Conclusions

We consider the contributions of this study to be conceptual in addition to empirical and hope that its framework will inspire further consideration of diversity gradients that aims to integrate ecological and evolutionary mechanisms across scales. Our global hierarchical approach represents an analytical paradigm shift away from the traditional analysis of fine-scale assemblages as independent spatial units. But there are obvious limits to our analysis. While the strong association of vertebrates with dominant vegetation types and the observed biotic independence of bioregions support their delineation as major evolutionary arenas, challenges remain surrounding the demarcation of the exact boundaries of such regions, the accuracy of past climate reconstructions, and their comparability across clades. Future availability of higher resolution phylogenies of the four vertebrate clades will allow more rigorous comparative approaches within and across lineages, but even comprehensive, strongly supported phylogenetic reconstructions are unlikely to provide vital information regarding the estimation of ancestral distributions (or ranges) and extinction rates [69]. Thus, our model can be viewed as a template on top of which other processes surely influence the origin and maintenance of diversity. For example, glaciation cycles influence speciation and extinction rates [36] and play an important role in driving recent speciation over broad scales [70]. Historical climate dynamics along elevation gradients in particular are known to create opportunities for rapid climate-associated parapatric or allopatric speciation and contribute strongly to the high richness of many tropical mountain areas [71]–[73]. Furthermore, a multitude of trophic interactions are likely to interact with these large-scale processes to cause positive, coevolutionary feedback loops, thus further increasing fine-scale and regional diversity [15].

Our findings show that energy availability has a large effect on both the regional pool and local sorting of richness. This highlights its importance for both evolutionary and ecological processes and the critical need to integrate these effects. This is especially crucial today, given the attention paid to recent models predicting the effects of climate change on the richness of whole gridded assemblages. The redundancy of information and conflation of ecological and evolutionary processes in smaller scale models impede interpretation in a way that is overcome in our analysis. Here we have shown how history can be integrated into a model predicting diversity with area, productivity, and temperature at the global scale. The separate consideration of drivers of diversification and finer scale occupancy and their joint effects on observed gradients of species richness should help pave the way for a more integrated macro-evolutionary and -ecological understanding of the origin and maintenance of global richness gradients.

## Materials and Methods

### Bioregion Selection, Time-Integrated Area Calculations

We selected 32 well-established, geographically and climatically distinct bioregions (Figure 1). These bioregions correspond to the biomes (tundra, desert, grassland, boreal forest, temperate forest, tropical moist/wet forest, tropical dry forest/savanna, and Mediterranean forest/shrublands) within the world's main biogeographic realms (Neartic, Paleartic, Neotropical, Australian, IndoMalayan, and Afrotropics) as described by Olson et al. [46] and also used in the Wildfinder vertebrate distribution database (see below) [74]. Although we do not have detailed, fine-scale records throughout every interval of time for the past 55 million years, enough information exists regarding the age of all biomes and directionality of their expansion and contraction to make reasonable estimates of the measures of their area integrated over time (Table S5). We excluded the “Mangroves” biome (Biome ID 14 in [46],[74]) and also the “Montane Grasslands & Shrublands” Biome (Biome ID 10 in [46],[74]). The latter was not included due to the difficulty in estimating areal and climate changes over their steep gradients over such a long time period. For example, in the Andes, different biomes occur at different elevations on the western and eastern slopes at different latitudes, and the available data are not sufficient to accurately estimate the elevations of the southern, central, and northern Andes at various time intervals since the Miocene, as each chain has uplifted at different rates and at different times [75]. This is critical information to be able to reconstruct the areal extent over time of each bioregion in the Andes and a general problem common to all of the world's mountain ranges, which is why they were excluded from our analysis.

The last 55 million years is an appropriate interval of time to measure the time-integrated area of the world's biomes within realms for two reasons. First, the beginning of the window of time is 10 million years after the massive extinction, which occurred 65 million years ago, causing major upheaval in the vertebrates. By 55 million years ago, the biosphere had recovered but its biota was very different from the plants and animals that had dominated the Cretaceous. Second, most of the “higher taxa”—that is, ancestors of modern lineages of vertebrates that now dominate the extant diversity of mammals, birds, amphibians, and reptiles (for example, fossils recognizable as extant genera)—are already represented in the fossil record by 55 million years ago [76]. Plant communities by the Eocene are, for the first time, composed of Angiosperms and Gymnosperms that are recognizable as the “genera” and “families” that are dominant in today's biomes [62],[77]. Thus, the biota in the Eocene has a “modern aspect” [76],[77].

The Earth's biomes have experienced large changes over the last 55 million years due to the consistent pattern of cooling and drying that has steadily taken place over this period of time [62],[78],[79]. Average global temperatures have plummeted from 27°C 55 million years ago to today's average of 15°C and precipitation has similarly dropped [62],[80]. For the moist/wet forest biomes (boreal forest, temperate forest, and tropical moist/wet forest) we used maps generated by Fine and Ree (2006) that were based on five sources: [49],[62],[81]–[83]. For the other biomes, our approach to estimate the time-integrated area of each biome was first to try to determine the paleobotanical consensus opinion for the age of each biome (Table S5). Then, we took the extant area of that biome and backcasted in time over the years that it has been present, reasoning that as tropical forests have receded during the past 55 MY years, dry and cold biomes such as tundra, desert, Mediterranean, grassland, and dry forest/savanna must have increased in size from the date of their origin to today's area.

We made two interpretations—a “wet” and a “dry” interpretation (Table S5). These two interpretations span the diverse opinions regarding the extent and age of the world's biomes over the last 55 million years and thus gauge the robustness of our results according to a range of expert opinions. For example, desert plants are absent in fossil records until about 2 Ma [77], even though it is hypothesized with molecular dating that plant lineages today found only in desert floras are at least 50 Ma old [62]. Thus, the consensus opinion is that deserts were probably present in the Eocene, but much restricted in size compared to today. For example, evaporite sediments point to extreme aridity in western Africa, Arabia, and central Asia in the late Miocene [82]. We thus made two estimates for the time-integrated area of desert biomes. The “wet” interpretation gives deserts an origin of 34 MYA but covering 10% of their current area from 34 MYA until 2 MYA, which is consistent with the lack of fossil evidence for any desert plant communities. The “dry” interpretation also gives the origin of deserts 34 MYA but has deserts covering the same areal extent as today since their origin, which is almost certainly an overestimate but is possible given the ancient age of some desert plant lineages and the difficulty of fossilization of desert environments (Table S5).

Our wet and dry interpretations both yield qualitatively similar results, and for simplicity, we focus on the “wet” interpretation throughout the article. The current-day extent of a bioregion as given in [46] yielded our predictor variable *Area* (units km^2^). Time-integrated area (*TimeArea*, in units year km^2^) was given as the integrated areal extent of a bioregion over 55 million years, or simply the sum of the area estimated for each of the 55 one-million-year periods. We acknowledge that this offers only a first order approximation. While exact values will be subject to change as paleoecological knowledge advances, we expect these changes to refine the details rather than radically alter overall patterns, which would have relatively little effect on our analyses, and thus we do not expect systematic biases in our results.

While topographic heterogeneity is expected to also influence the potential for reproductive isolation [32], in this dataset (which excludes montane regions) it is largely captured by bioregion *Area* and does not yield improved predictions (see Table S12).

### Bioregion Species Data

We aggregated existing eco-regional terrestrial vertebrate species lists for the selected 32 bioregions from the Wildfinder distribution database [74]. We excluded all eco-regions in biomes not selected for analysis (see above), including all montane eco-regions (which have a total of 1,015 terrestrial vertebrate species restricted to them). This resulted in 54,122 bioregion occurrence records for 25,696 species (9,229 birds, 4,607 amphibians, 4,631 mammals, and 7,229 reptiles). We calculated terrestrial vertebrate richness (“vertebrate richness”) per bioregion in three different ways: *Total*, which includes every vertebrate species found within each bioregion; *Resident*, which only counts species in the bioregion with the largest proportion of its geographic range; and *Endemic*, which counts only species that are restricted to a single bioregion (see Table S2 for complete raw data). Assigning each species only to its dominant bioregion to eliminate pseudo-replication yields a *Resident* richness pattern very similar to that of *Total* richness (*r*_S_ = 0.85, Table S4). For the analyses, vertebrates were divided into ectotherms (amphibians and reptiles) and endotherms (birds and mammals) and further separated into birds, mammals, reptiles, and amphibians. All richness values were natural log-transformed.

### Finer Scale Species Data

Species occurrence data across grid cells were compiled from global expert opinion range maps extracted across a 110×110 km equal area grid in a Behrman projection. For mammals [84], and amphibians, sources were the IUCN assessment (http://www.iucnredlist.org). For birds, breeding distributions were compiled from the best available sources for a given broad geographical region or taxonomic group [85]. For reptiles, global-scale expert range maps have not yet been compiled, and they were therefore not included in the grid cell assemblage analyses. We excluded all cells that were not >50% inside the selected bioregion boundaries as described above (and shown in Figure 1). Only cells with >50% dry land and with at least one species from each of the three vertebrate groups were included in the analysis, resulting in 9,253 cells. For each grid cell we summarized richness of *Resident* species (i.e., species were counted if they occurred in several grid cells only within the same bioregion) and of *Total* species (i.e., species were counted whether they occurred in multiple grid cells within the same or in a different bioregion). Values were log10-transformed before analysis. For *Total* species, the full database consisted of a total of 2,966,137 grid cell records (birds 2,010,091; mammals 695,133; and amphibians 260,913).

### Bioregion and Finer Scale Environmental Data

Bioregion-typical temperature estimates (*Temperature*) were based on average annual temperatures calculated from the University of East Anglia's Climatic Research Unit gridded climatology 1961–1990 dataset at native 10-min resolution [86]. For estimates of bioregion-typical annual net primary productivity, we used an average from 17 global models at a spatial resolution of 0.5 degrees latitude-longitude [87]. Average bioregion productivity (*Productivity*, units grams Carbon m^−2^ year^−1^) was calculated from all 0.5×0.5 degree grid cells that predominantly fall inside a bioregion, and summed productivity (*AreaProductivity*, units grams Carbon year^−1^) was then given by the product of this value and bioregion *Area*. With bioregions defined by their typical environmental conditions, we assumed average productivity characteristic of a bioregion to have been constant through time [49],[62]. Time-integrated productivity (*TimeAreaProductivity*, unit grams Carbon) was thus given as the product of *Productivity* and *TimeArea*. Values for all bioregion predictor variables are given in Table S1. All response and predictor variables were natural log-transformed for analysis, except for temperature, which was 1/kT transformed (where k is the Boltzmann constant, see [44]). We used the same global net primary productivity dataset [87] to estimate productivity at the level of 110×110 km grid cells. First, we calculated average grid cell productivity (*NPP*) across all encompassing 0.5×0.5 degree grid cells. Second, we normalized each grid cell by dividing by the maximum productivity grid cell value observed in a bioregion, resulting in a measure of proportional productivity (*PropNPP*) varying from 0 to 1.

### Bioregion Analyses

We performed a total of nine GLM models on the bioregion data and used the Akaike criterion to identify those offering the best fit [88]. Six models were given in the form of single predictors (*Temperature*, *Area*, *Productivity*, *AreaProductivity*, *TimeArea*, and *TimeAreaProductivity*). An additional three models were formed by the combination of the latter three variables with *Temperature*. We performed a separate set of analyses to assess the potential additional effect of elevation range within a bioregion, but adding this variable to any of the three two-predictor models did not improve model fit, and thus we excluded the variable from further consideration. Because of the strong independence of sampling units both in terms of response (no overlap in species) and predictor variables (by definition each bioregion is environmentally highly distinct from neighboring bioregions), the usual concerns about spatial autocorrelation affecting model results [89],[90] do not apply to this analysis, and additional spatial regression analysis was not performed.

### Finer Scale Analyses

Having established models of bioregion richness, we assessed the success of predictions of resident bioregional richness to explain the species richness (*Total* and *Resident*, see above) of all 110×110 km grid cells within bioregions (for a conceptual overview of the analytical steps, see Figure 3). Note that unlike the bioregional tests described above, analyses at this scale do double-count species. In our study we make the simplifying assumption that diversification processes are sufficiently accounted for at the bioregional scale. The models at the within-bioregion scale then address the sorting of these species each into multiple grid cells, with multiple occurrences an integral part of the signal. We acknowledge that, depending on taxon and region, diversification processes may still exert influence on the within-bioregion patterns of distribution and richness, and we hope that our work will spur further research into additional approaches that can be integrated across all scales.

We first evaluated bioregion predicted resident richness alone (in essence testing for a random sorting of bioregion species into finer scale assemblages), then included bioregion *Area* as an additional predictor, and finally we added estimates of grid cell NPP as a finer scale predictor. We first performed simple GLM models with all 9,253 grid cells as sampling units, together with bioregion *Resident* richness as predicted by the *TimeAreaProductivity*+*Temperature* and *AreaProductivity*+*Temperature* models as a first predictor (*BioregPred*) and bioregion *Area* as a second predictor (Figure 3, Table S9). In the same GLM we then added grid cell proportional net primary productivity (*CellPropNPP*, i.e., relative productivity within a bioregion, see above) as an additional predictor. In preliminary post hoc analyses with a number of environmental variables *CellPropNPP* remained by far the strongest, in line with recent work on within-regional richness filters that also find productivity-related variables to be dominant [26],[27]. Given the nested nature of these analyses we focus on pseudo-*r* ^2^ values (fit of observed versus predicted) and visual examination of results in the form of partial residual plots (Figure 3). For this first demonstration, focused on a single variable, we did not include further analyses additionally fitting the signal of spatial autocorrelation.

We performed a second set of analyses in an explicit mixed effects model setting (Table S10), with bioregion as a random effect (R library lme4, Version 0.999375-32, function lmer). As in the GLM model, grid cell richness is first fitted by the predictions for regional resident species richness (*BioregPred*, see Table 1), and then by area of the region (*Area*), and grid-cell-level NPP (*NPP*). Region was fitted as a random effect, and the slope and strength of *BioregPred* and *BioregPred*+*Area* as fixed effects were assessed (model formula in R: lmer (y∼*BioregPred*+*Area*+(1|*Bioregion*)). The additional effect of grid cell *NPP* was then evaluated by fitting it as an additional fixed effect with a globally constant slope (NPP_const_) and by allowing the NPP–richness relationship to vary within regions as random slope (NPP_var_) (model formula in R: lmer (y∼*BioregPred*+*Area*+(1|*Bioregion*)+(NPP|*Bioregion*)).

### Data Deposition

The data are deposited in the Dryad Repository (http://dx.doi.org/10.5061/dryad.45672js4).

## Supporting Information

Figure S1 Bioregion independence at different taxonomic ranks. The frequency of Jaccard similarity values is shown ([count of shared taxa]/[count of taxa in both]) expressed in % (Jaccard * 100) for all bioregion combinations (*N* = 496) for different taxonomic ranks. Results confirm high independence of bioregions at the species and genus rank and moderate independence at family rank. See also Table S3.
(TIF)

Figure S2 Partial residual plots for the joint effects *TimeAreaProductivity* and *Temperature* on *Resident* species richness (ln-transformed). Partial residual plots illustrate the relationship between a predictor and the response given other predictors in the model. Specifically, this is a plot of r_i_+bx_i_ versus x_i_, where r_i_ is the ordinary residual for the i-th observation, x_i_ is the i-th observation, and b is the regression coefficient estimate. Colors indicate biome membership (see Figure 1 for legend). For detailed model results, see Table S7.
(TIF)

Figure S3 Partial residual plots for the joint effects of *TimeAreaProductivity* and *Temperature* on *Endemic* species richness (ln-transformed). Partial residual plots illustrate the relationship between a predictor and the response given other predictors in the model. For other details, see Figure S2.
(TIF)

Figure S4 Partial residual plots for the variation in grid cell richness of *Resident* species among bioregions. In this model *Resident* richness is predicted by the bioregion (TimeAreaProductivity+Temperature) model and bioregion current-day *Area*. Partial residual plots illustrate the relationship between a predictor and the response given other predictors in the model. Specifically, this is a plot of r_i_+bx_i_ versus x_i_, where r_i_ is the ordinary residual for the i-th observation, x_i_ is the i-th observation, and b is the regression coefficient estimate. Colors indicate biome membership (see Figure 1 for color legend and Figure 3 for results without *Area*).
(TIF)

Table S1 List of bioregions and predictor variables in the analysis. *TimeArea* and *TimeAreaProductivity* values are from integration of bioregion area over 55 million years. For further details and geographic locations, see Figure 1.
(DOC)

Table S2 Bioregion species richness values. *Total*: includes all species with ranges extending into a given bioregion (many species represented several times in different bioregions). *Resident*: includes only species with the greatest portion of their range extending into a given bioregion (each species is represented only once). *Endemic*: includes only species with no portion of range extending beyond a given bioregion. Vert., Vertebrates (Birds+Mammals+Amphibians+Reptiles). Amph., Amphibians.
(DOC)

Table S3 Median Jaccard similarity (%) of bioregion composition at three different taxonomic ranks. Jaccard similarity is given as ([count of shared taxa]/[count of taxa in both]) expressed in % (Jaccard * 100). For a given bioregion and taxon, values are medians from the comparison with all 31 other regions, respectively. See also Figure S1.
(DOC)

Table S4 Spearman rank correlations among bioregion species richness values for *Total*, *Resident*, and *Endemic* categories for all vertebrates, endotherms, and ectotherms, and each vertebrate clade separately (*N* = 32 bioregions). For richness definitions see Table S2.
(DOC)

Table S5 Details regarding the ages of biomes and the sources consulted in order to calculate the area over time for each of the world's bioregions.
(DOC)

Table S6 Spearman rank correlations of predictor variables among bioregions (N = 32).
(DOC)

Table S7 Predictors of bioregion richness for Endotherms (mammals+birds) and Ectotherms (amphibians+reptiles) with details on slope estimates. Species richness values and all predictors except temperature were ln-transformed; temperature is given as 1/kT (where k is the Boltzmann constant). For other details see Table 1.
(DOC)

Table S8 Predictors of bioregion richness. Results for all taxa. For other details see Table 1.
(DOC)

Table S9 Comparison of AIC values of alternative formulations of models combining the effects of *TimeArea* and *Productivity*, and of *TimeArea*, *Productivity*, and *Temperature* on bioregion species richness. *TimeArea* and *Productivity* are either integrated into a single variable (*TimeAreaProductivity*, see Figure 1), modeled additively, or modeled as an interaction. Models with >3 units AIC larger than the model with the smallest AIC within a group (i.e., significantly worse) are marked in bold.
(DOC)

Table S10 Comparison of AIC values of *TopoRange* (log(maximum − minimum elevation) in a bioregion) as an alternative predictor of bioregion species richness. *Null* model is fitting the intercept only. The variable *Area*, which is correlated with *TopoRange* (r_Spearman_ = 0.58, *N* = 32), offers either equal or better fit.
(DOC)

Table S11 Spearman rank correlations among *Total*, *Resident*, and *Endemic* richness for different taxa across 110 km quadrants (*N* = 9,253). For richness definitions, see Table S2.
(DOC)

Table S12 Prediction success (*r* ^2^) of bioregion-level models of *Total* and *Resident* richness of 110 km grid cell assemblages (*N* = 9,253) based on general linear models. Grid cell richness is first fitted by the predictions for *Resident* species richness (“[Bioregion] Predicted richness,” see Table 1), and then additionally by *Area* of the bioregion, and grid-cell-level relative productivity (*CellPropProductivity*, calculated as proportion of maximum grid cell productivity in the region). Pseudo-*r* ^2^ values of observed versus fitted are listed.
(DOC)

Table S13 Prediction success of bioregion-level models of *Total* and *Resident* richness of 110 km grid cell assemblages (*N* = 9,253) based on mixed effects models. Grid cell richness is first fitted by the predictions for *Resident* species richness (“[Bioregion] Predicted richness,” see Table 1) and then additionally by bioregion *Area*, and grid-cell-level relative productivity (*CellPropProductivity*). Bioregion is fitted as a random effect, and the slope and strength of “[Bioregion] Predicted richness” and “[Bioregion] Predicted richness+*Area*” as fixed effects are assessed. Pseudo-*r* ^2^ values of observed versus fitted are listed. The additional effect of grid cell productivity was evaluated by fitting it as additional fixed effect with a globally constant slope (*CellPropProductivity*) and by allowing the relationship with richness to vary within regions as a random slope (*CellPropProductivity*_var_).
(DOC)

## Abbreviations

MY: million years
mya: million years ago
