# Supplementary figures and images for: Global Gradients in Vertebrate Diversity Predicted by Historical Area-Productivity Dynamics and Contemporary Environment
Source: PLoS Biol. 2012 Mar 27;10(3):e1001292. doi: 10.1371/journal.pbio.1001292 (PMC3313913; doi:10.1371/journal.pbio.1001292)

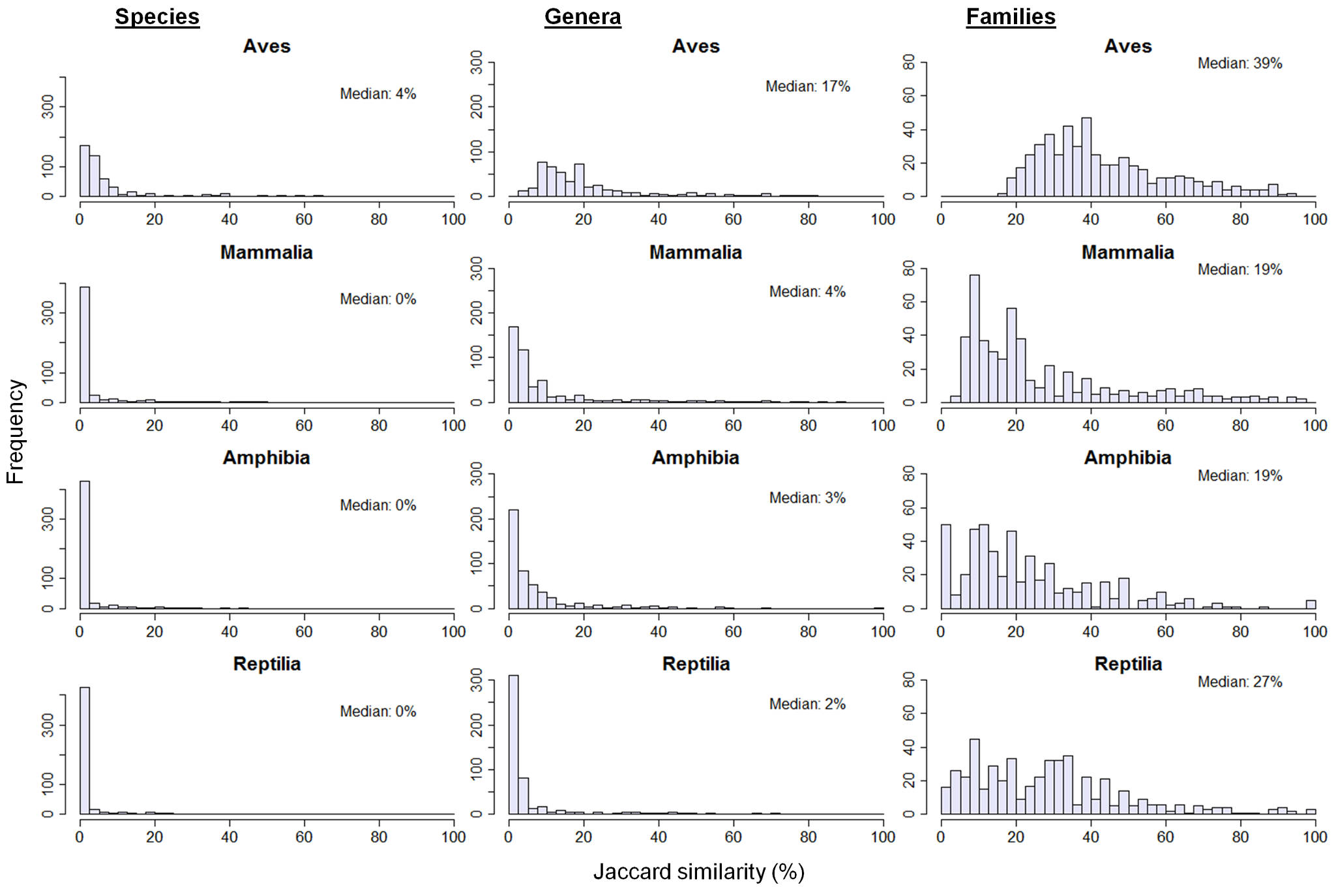

Supplement: Figure S1 — Bioregion independence at different taxonomic ranks. The frequency of Jaccard similarity values is shown ([count of shared taxa]/[count of taxa in both]) expressed in % (Jaccard * 100) for all bioregion combinations (N = 496) for different taxonomic ranks. Results confirm high independence of bioregions at the species and genus rank and moderate independence at family rank. See also Table S3. (TIF) [file pbio.1001292.s001.tif]

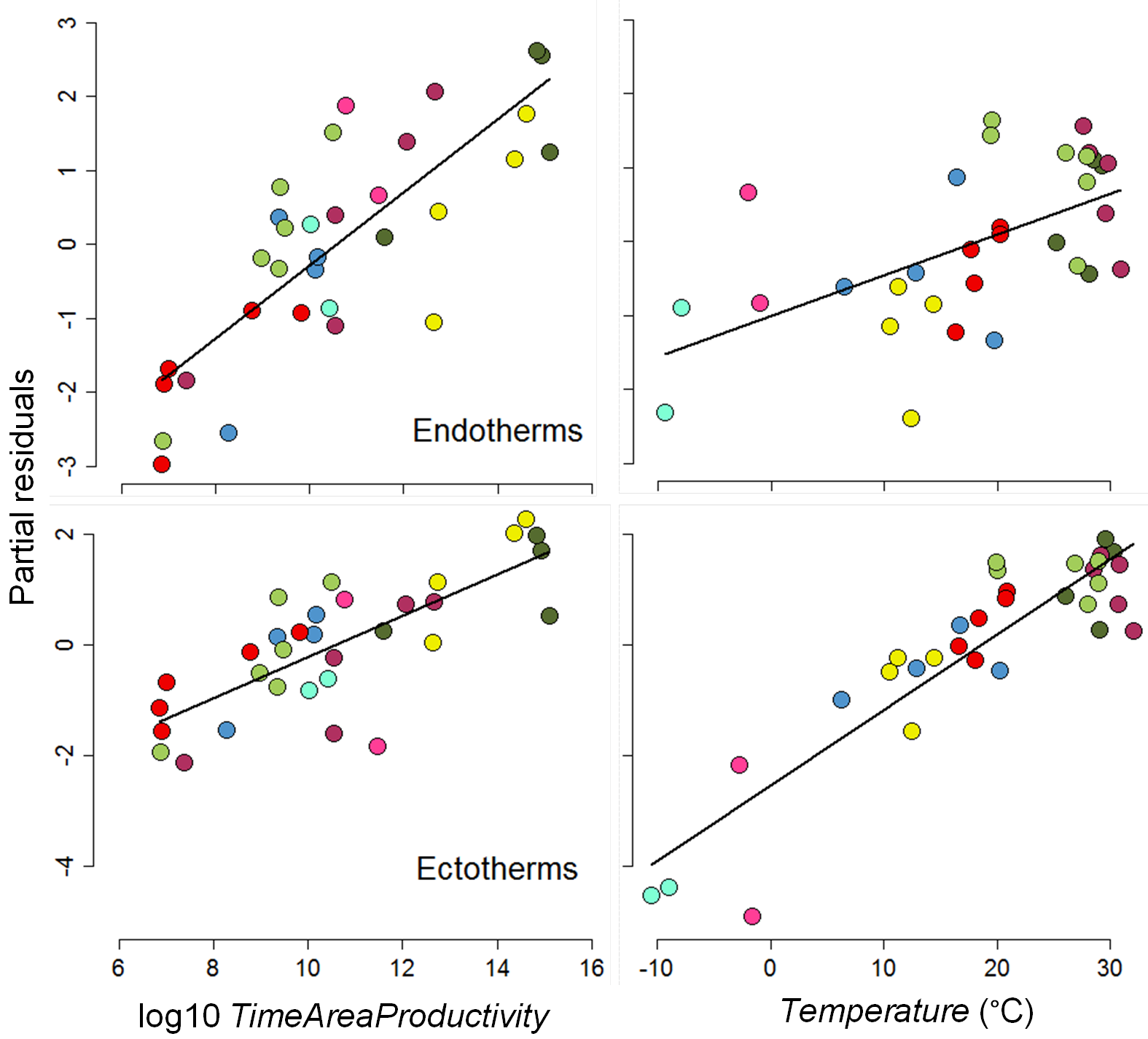

Supplement: Figure S2 — Partial residual plots for the joint effects TimeAreaProductivity and Temperature on Resident species richness (ln-transformed). Partial residual plots illustrate the relationship between a predictor and the response given other predictors in the model. Specifically, this is a plot of ri+bxi versus xi, where ri is the ordinary residual for the i-th observation, xi is the i-th observation, and b is the regression coefficient estimate. Colors indicate biome membership (see Figure 1 for legend). For detailed model results, see Table S7. (TIF) [file pbio.1001292.s002.tif]

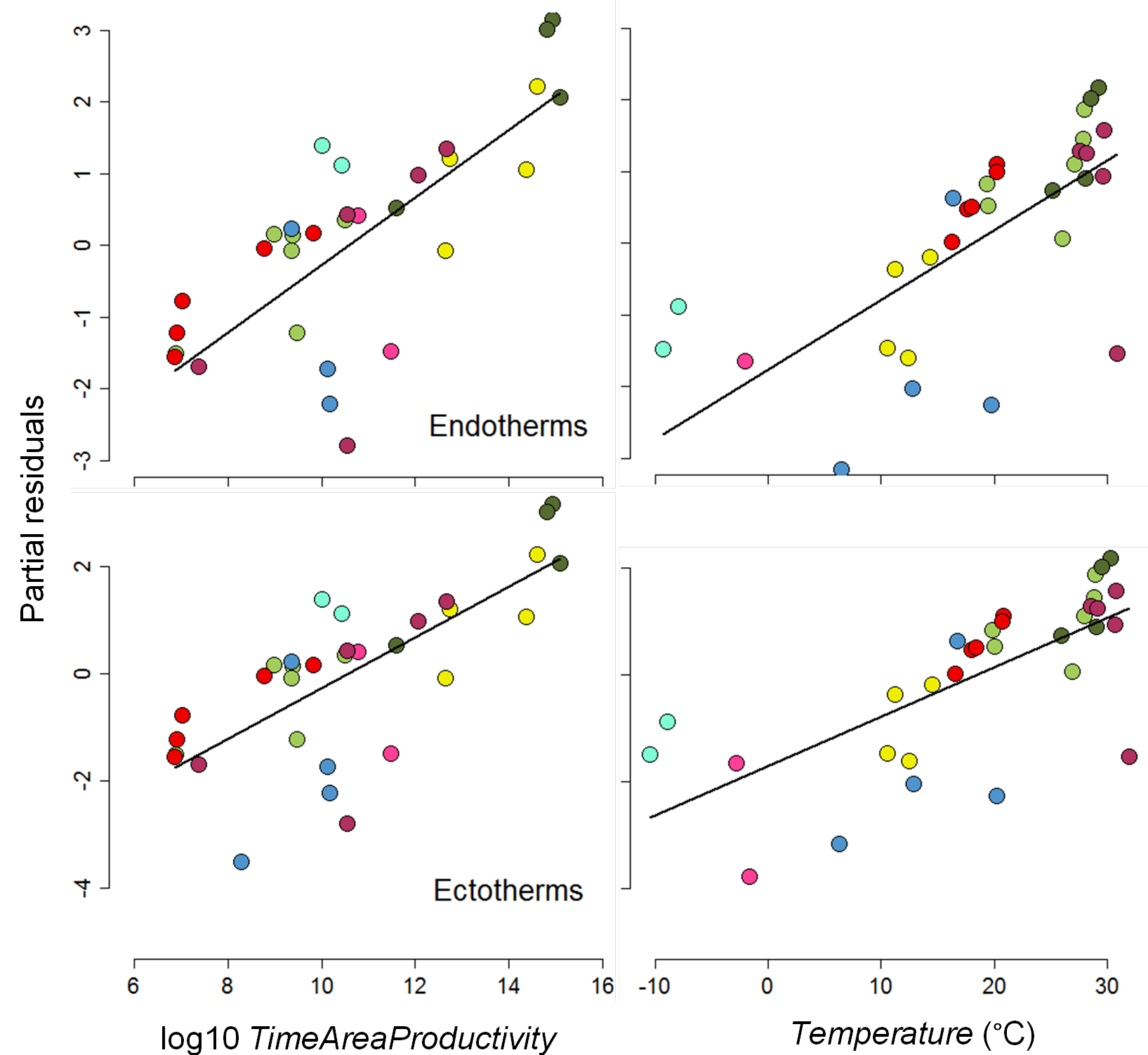

Supplement: Figure S3 — Partial residual plots for the joint effects of TimeAreaProductivity and Temperature on Endemic species richness (ln-transformed). Partial residual plots illustrate the relationship between a predictor and the response given other predictors in the model. For other details, see Figure S2. (TIF) [file pbio.1001292.s003.tif]

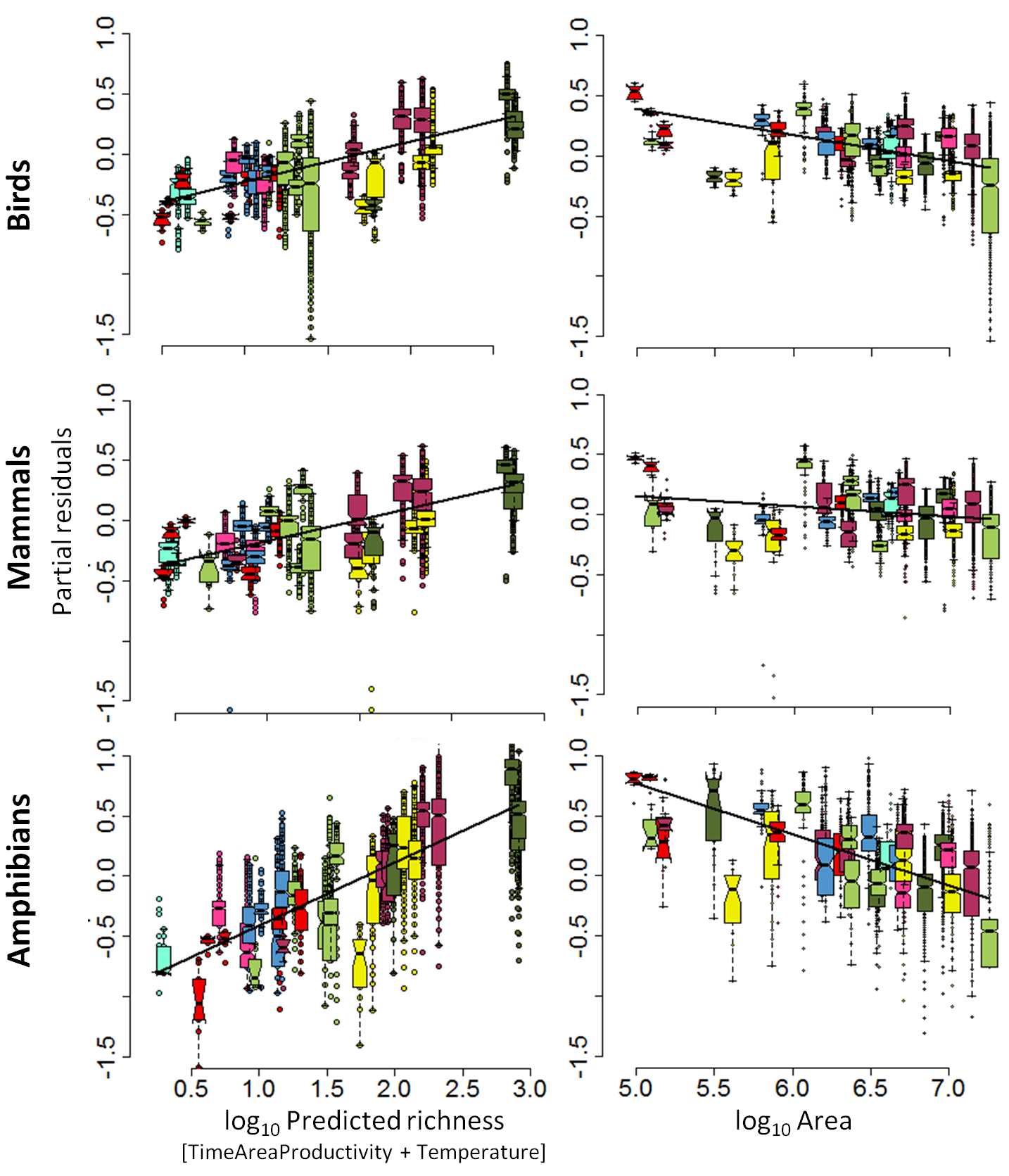

Supplement: Figure S4 — Partial residual plots for the variation in grid cell richness of Resident species among bioregions. In this model Resident richness is predicted by the bioregion (TimeAreaProductivity+Temperature) model and bioregion current-day Area. Partial residual plots illustrate the relationship between a predictor and the response given other predictors in the model. Specifically, this is a plot of ri+bxi versus xi, where ri is the ordinary residual for the i-th observation, xi is the i-th observation, and b is the regression coefficient estimate. Colors indicate biome membership (see Figure 1 for color legend and Figure 3 for results without Area). (TIF) [file pbio.1001292.s004.tif]
