# Supplementary material for: Global Gradients in Vertebrate Diversity Predicted by Historical Area-Productivity Dynamics and Contemporary Environment
Source: PLoS Biol. 2012 Mar 27;10(3):e1001292. doi: 10.1371/journal.pbio.1001292 (PMC3313913; doi:10.1371/journal.pbio.1001292)
Supplement: Table S1 — List of bioregions and predictor variables in the analysis. TimeArea and TimeAreaProductivity values are from integration of bioregion area over 55 million years. For further details and geographic locations, see Figure 1. (DOC) [file pbio.1001292.s005.doc]

**Table S1: List of bioregions and predictor variables in the analysis.** *TimeArea* and *TimeAreaProductivity* values are from integration of bioregion area over 55 million years. For further details and geographic locations see Fig. 1.
