# Supplementary material for: Global Gradients in Vertebrate Diversity Predicted by Historical Area-Productivity Dynamics and Contemporary Environment
Source: PLoS Biol. 2012 Mar 27;10(3):e1001292. doi: 10.1371/journal.pbio.1001292 (PMC3313913; doi:10.1371/journal.pbio.1001292)
Supplement: Table S2 — Bioregion species richness values. Total: includes all species with ranges extending into a given bioregion (many species represented several times in different bioregions). Resident: includes only species with the greatest portion of their range extending into a given bioregion (each species is represented only once). Endemic: includes only species with no portion of range extending beyond a given bioregion. Vert., Vertebrates (Birds+Mammals+Amphibians+Reptiles). Amph., Amphibians. (DOC) [file pbio.1001292.s006.doc]

**Table S2: Bioregion species richness values.** *All*: includes all species with ranges extending into a given bioregion (many species represented several times in different bioregions). *Resident*: includes only species with the greatest portion of their range extending into a given bioregion (each species represented only once). *Endemic*: includes only species with no portion of range extending beyond a given bioregion. Vert. – Vertebrates (Birds + Mammals + Amphibians + Reptiles). Amph. – Amphibians.
