# Supplementary material for: Global Gradients in Vertebrate Diversity Predicted by Historical Area-Productivity Dynamics and Contemporary Environment
Source: PLoS Biol. 2012 Mar 27;10(3):e1001292. doi: 10.1371/journal.pbio.1001292 (PMC3313913; doi:10.1371/journal.pbio.1001292)
Supplement: Table S3 — Median Jaccard similarity (%) of bioregion composition at three different taxonomic ranks. Jaccard similarity is given as ([count of shared taxa]/[count of taxa in both]) expressed in % (Jaccard * 100). For a given bioregion and taxon, values are medians from the comparison with all 31 other regions, respectively. See also Figure S1. (DOC) [file pbio.1001292.s007.doc]

**Table S3: Median Jaccard similarity (%) of bioregion composition at three different taxonomic levels.** Jaccard similarity is given as ([count of shared taxa] / [ count of taxa in both]) expressed in % (Jaccard *100). For a given bioregion and taxon, values are medians from the comparison with all 31 other regions, respectively. See also Fig. S1.
