# Supplementary material for: Global Gradients in Vertebrate Diversity Predicted by Historical Area-Productivity Dynamics and Contemporary Environment
Source: PLoS Biol. 2012 Mar 27;10(3):e1001292. doi: 10.1371/journal.pbio.1001292 (PMC3313913; doi:10.1371/journal.pbio.1001292)
Supplement: Table S4 — Spearman rank correlations among bioregion species richness values for Total, Resident, and Endemic categories for all vertebrates, endotherms, and ectotherms, and each vertebrate clade separately (N = 32 bioregions). For richness definitions see Table S2. (DOC) [file pbio.1001292.s008.doc]

**Table S4: Spearman rank correlations among bioregion species richness values** for *Total*, *Resident* and *Endemic* categories for all vertebrates, endotherms and ectotherms, and each vertebrate clade separately (N = 32). For richness definitions see Table S2.
