# Supplementary material for: Global Gradients in Vertebrate Diversity Predicted by Historical Area-Productivity Dynamics and Contemporary Environment
Source: PLoS Biol. 2012 Mar 27;10(3):e1001292. doi: 10.1371/journal.pbio.1001292 (PMC3313913; doi:10.1371/journal.pbio.1001292)
Supplement: Table S5 — Details regarding the ages of biomes and the sources consulted in order to calculate the area over time for each of the world's bioregions. (DOC) [file pbio.1001292.s009.doc]

**Table S5: Details regarding the ages of biomes and the sources consulted in order to calculate the area over time for each of the world’s bioregions.**

| **Biome** | **Age of Origin** | **Comments** | **“Wet Interpretation”** | **“Dry Interpretation”** | **Sources** |
| --- | --- | --- | --- | --- | --- |
| Tundra | 5-10 million years | Estimates of arctic climates in the Miocene suggest a 10 MYA origin of tundra vegetation. However, there is little fossil evidence to support this early date (Truswell 1990) as the first unequivocal fossil tundra vegetation dates from 2-3 Ma. | We conservatively take current tundra area and extend it into the past for 11.2 million years. | We conservatively take current tundra area and extend it into the past for 11.2 million years. | (Wolfe 1985, Truswell 1990, Willis and McElwain 2002) |
| Boreal Forests | 5-10 million years | Very recent origin. See Fine and Ree (Fine and Ree 2006) for detailed justification and explanation. | “Interpretation 5” from Fine and Ree 2006, based primarily from Willis and McElwain (2002). | “Interpretation 1” from Fine and Ree (2006), based primarily from (Beerling and Woodward 2001). |  |
| Temperate Moist Forests | Older than Eocene | See Fine and Ree (2006) for detailed justification and explanation. | “Interpretation 5” from Fine and Ree 2006, based primarily from Willis and McElwain (2002). | “Interpretation 1” from Fine and Ree (2006), based primarily from (Beerling and Woodward 2001). |  |
| Temperate grasslands | 25 million years | Grass lineages (Poaceae) are older than 50 MYA but pollen records suggest that grasses were relatively rare and did not cover substantial areas of the earth until 25 million years ago. For example, grass pollen accounts for less than 1/100 of total pollen abundance in many samples between 55 and 40 Ma, but by around 25 Ma, palynologists report a large increase in pollen abundance. Yet massive expansion of grasslands occurred only in the past few million years. | Sets origin at 25 MYA, with reduced area of grassland (10% of current values) from 25 MYA until 2 MYA, and then for the past 2 million years, same area as today’s values. | Today’s area from 25 MYA to the present. | (Janis 1993, Webb et al. 1995, Jacobs et al. 1999, Willis and McElwain 2002) |
| Mediterranean shrublands | 10 million years. | Dry Mediterranean-type sclerophyllous vegetation that looks similar to what we find in Mediterranean biomes today do not appear in the fossil recorduntil the Miocene (10 ma). | We set the origin of Mediterranean biome at 11.2 MYA with steady increase from 0 until today's area. | Today’s area from 34 MYA to the present. | (Janis 1993, Willis and McElwain 2002) |
| Deserts | Probably as old as 50 MYA, but only covered small areas until the last few million years. | Desert plants are absent in fossil record until about 2 Ma, even though it is hypothesized with molecular dating that plant lineages today found only in desert floras are at least 50 Ma old. Many drought adapted shrubs became established from 40 Ma. Deserts were probably present in the Eocene, but much restricted in size compared to today. Evaporite sediments point to extreme aridity in western Africa, Arabia and central Asia in the late Miocene. | We set the age of deserts at 34 million years, and allow them to cover 10% of their current extent from 34-2 MYA, and from 2 MYA until the present they cover their current extent. | We set the age of deserts at 34 MYA and they cover the same area as today for the entire time period. | (Willis and McElwain 2002) |
| Tropical savanna and dry forests | Probably older than 55 million years, but covered only small areas. | Tropical woody savanna and dry forest, are probably older than Eocene (Graham 1999), , but there exists no unequivocal fossil evidence until the mid Miocene. Then, according to pollen records and macrofossils, there was a large increase in dry forest from the Miocene towards the present (Burnham 1995, Burnham and Carranco 2004, Pennington et al. 2006).  There are fossils of woody savanna vegetation from 40 Ma in the Great Plains of NAm (Retallack 1992). But these plant communities may have existed as early as 50 Ma in arid continental interiors (but only covering small areas, Janis 1993). Acacias and Eucalypts, typical dry forest and woddy savanna elements are dated at 38-24 Ma. Nevertheless, tropical savannas are not thought to cover large areas until 8 Ma or sooner, (Cerling et al. 1997, Beerling and Osborne 2006). | We set the origin of dry forests at 11.2 MYA and they cover the same area as today for the entire time period. | We set the age of dry forests at 34 MYA and they cover the same area as today for the entire time period. | (Retallack 1992, Janis 1993, Burnham 1995, Burnham and Carranco 2004, Pennington et al. 2006). |
| Tropical moist/wet forests | Older than 55 million years. | Tropical rain forests covered much larger areas on the planet in the Eocene and Miocene. In the last several million years, these forests have retreated to the equatorial areas. | “Interpretation 5” from Fine and Ree (2006), based primarily from Willis and McElwain (2002). | “Interpretation 1” from Fine and Ree (2006), based primarily from (Beerling and Woodward 2001). |  |

**Table S5 references:**

Beerling, D. and F. Woodward. 2001. Vegetation and the terrestrial carbon cycle: modelling the first 400 million years. Cambridge Univ Pr.

Beerling, D. J. and C. P. Osborne. 2006. The origin of the savanna biome. Global Change Biology **12**:2023-2031.

Burnham, R. 1995. A new species of winged fruit from the Miocene of Ecuador: Tipuana ecuatoriana (Leguminosae). American Journal of Botany **82**:1599-1607.

Burnham, R. and N. Carranco. 2004. Miocene winged fruits of Loxopterygium (Anacardiaceae) from the Ecuadorian Andes. American Journal of Botany **91**:1767.

Cerling, T., J. Harris, B. MacFadden, M. Leakey, J. Quade, V. Eisenmann, and J. Ehleringer. 1997. Global vegetation change through the Miocene/Pliocene boundary. Nature **389**:153-158.

Fine, P. V. A. and R. H. Ree. 2006. Evidence for a time-integrated species-area effect on the latitudinal gradient in tree diversity. American Naturalist **168**:796-804.

Graham, A. 1999. Late Cretaceous and Cenozoic history of North American vegetation, north of Mexico. Oxford University Press, New York.

Jacobs, B., J. Kingston, and L. Jacobs. 1999. The origin of grass-dominated ecosystems. Annals of the Missouri Botanical Garden **86**:590-643.

Janis, C. 1993. Tertiary mammal evolution in the context of changing climates, vegetation, and tectonic events. Annual Review of Ecology and Systematics **24**:467-500.

Pennington, R., C. Lewis, and J. Ratter. 2006. An overview of the plant diversity, biogeography and conservation of neotropical savannas and seasonally dry forests. Pages 1-29 *in* R. Pennington, C. Lewis, and J. Ratter, editors. Neotropical savannas and seasonlly dry forests: plant diversity, biogeography, and conservation. CRC Press, Florida.

Retallack, G. J. 1992. Paleosols and changes in climate across the Eocene/Oligoncene boundary. Pages 382-398 *in* D. R. Prothero and W. A. Berggren, editors. Eocene-Oligocene climatic and biotic evolution. Princeton University Press, Princeton.

Truswell, E. M. 1990. Cretaceous and Tertiary vegetation of Antarctica: a palynological perspective. Pages 71-88 *in* T. N. Taylor and E. L. Taylor, editors. Antarctic paleobiology. Springer, New York.

Webb, S. D., R. C. Hulbert, and W. D. Lambert. 1995. Climatic implications of large-herbivore distributions in the Miocene of North America. Pages 91-108 *in* E. S. Vrba, G. H. Denton, T. C. Patridge, and L. H. Burckle, editors. Paleoclimate and evolution. Yale University Press, New Haven.

Willis, K. and J. McElwain. 2002. The evolution of plants. Oxford University Press, Oxford.

Wolfe, J. A. 1985. Distribution of major vegetation types during the Tertiary.*in* E. T. Sundquist and W. S. Broecker, editors. The Carbon Cycle and Atmostpheric CO2: natural variations Archean to present. American Geophysical Union, Washington DC.
