# Supplementary material for: Global Gradients in Vertebrate Diversity Predicted by Historical Area-Productivity Dynamics and Contemporary Environment
Source: PLoS Biol. 2012 Mar 27;10(3):e1001292. doi: 10.1371/journal.pbio.1001292 (PMC3313913; doi:10.1371/journal.pbio.1001292)
Supplement: Table S6 — Spearman rank correlations of predictor variables among bioregions (N = 32). (DOC) [file pbio.1001292.s010.doc]

**Table S6: Details regarding the ages of biomes and the sources consulted in order to calculate the area over time for each of the world’s bioregions.**
