# Supplementary material for: Global Gradients in Vertebrate Diversity Predicted by Historical Area-Productivity Dynamics and Contemporary Environment
Source: PLoS Biol. 2012 Mar 27;10(3):e1001292. doi: 10.1371/journal.pbio.1001292 (PMC3313913; doi:10.1371/journal.pbio.1001292)
Supplement: Table S7 — Predictors of bioregion richness for Endotherms (mammals+birds) and Ectotherms (amphibians+reptiles) with details on slope estimates. Species richness values and all predictors except temperature were ln-transformed; temperature is given as 1/kT (where k is the Boltzmann constant). For other details see Table 1. (DOC) [file pbio.1001292.s011.doc]

**Table S7: Predictors of bioregion richness for Endotherms (mammals + birds) and Ectotherms (amphibians + reptiles) with details on slope estimates**. Species richness values and all predictors except temperature were ln transformed; temperature is given as 1/kT (where k is the Boltzmann constant). For other details see Table 1.
