# Supplementary material for: Global Gradients in Vertebrate Diversity Predicted by Historical Area-Productivity Dynamics and Contemporary Environment
Source: PLoS Biol. 2012 Mar 27;10(3):e1001292. doi: 10.1371/journal.pbio.1001292 (PMC3313913; doi:10.1371/journal.pbio.1001292)
Supplement: Table S8 — Predictors of bioregion richness. Results for all taxa. For other details see Table 1. (DOC) [file pbio.1001292.s012.doc]

**Table S8: Predictors of bioregion richness.** Results for all taxa. For other details see Table 1.
