# Supplementary material for: Global Gradients in Vertebrate Diversity Predicted by Historical Area-Productivity Dynamics and Contemporary Environment
Source: PLoS Biol. 2012 Mar 27;10(3):e1001292. doi: 10.1371/journal.pbio.1001292 (PMC3313913; doi:10.1371/journal.pbio.1001292)
Supplement: Table S9 — Comparison of AIC values of alternative formulations of models combining the effects of TimeArea and Productivity, and of TimeArea, Productivity, and Temperature on bioregion species richness. TimeArea and Productivity are either integrated into a single variable (TimeAreaProductivity, see Figure 1), modeled additively, or modeled as an interaction. Models with >3 units AIC larger than the model with the smallest AIC within a group (i.e., significantly worse) are marked in bold. (DOC) [file pbio.1001292.s013.doc]

**Table S9: Comparison of AIC values of alternative formulations of models** combining the effects of *TimeArea* and *Productivity*, and of *TimeArea*, *Productivity* and *Temperature* on bioregion species richness. *TimeArea* and *Productivity* are either integrated into a single variable (*TimeAreaProductivity*, see Fig 1), modeled additively, or as interaction. Models with > 3 units AIC larger than the model with smallest AIC within a group (i.e. significantly worse) are marked in bold.
