# Supplementary material for: Global Gradients in Vertebrate Diversity Predicted by Historical Area-Productivity Dynamics and Contemporary Environment
Source: PLoS Biol. 2012 Mar 27;10(3):e1001292. doi: 10.1371/journal.pbio.1001292 (PMC3313913; doi:10.1371/journal.pbio.1001292)
Supplement: Table S10 — Comparison of AIC values of TopoRange (log(maximum − minimum elevation) in a bioregion) as an alternative predictor of bioregion species richness. Null model is fitting the intercept only. The variable Area, which is correlated with TopoRange (rSpearman = 0.58, N = 32), offers either equal or better fit. (DOC) [file pbio.1001292.s014.doc]

**Table S10: Comparison of AIC values** of *TopoRange* (log(maximum - minimum elevation) in a bioregion) as an alternative predictor of bioregion species richness. *Null* model is fitting the intercept only. The variable *Area*, which is correlated with *TopoRange* (r_Spearman_ = 0.58, N = 32) offers either equal or better fit.
