# Supplementary material for: Global Gradients in Vertebrate Diversity Predicted by Historical Area-Productivity Dynamics and Contemporary Environment
Source: PLoS Biol. 2012 Mar 27;10(3):e1001292. doi: 10.1371/journal.pbio.1001292 (PMC3313913; doi:10.1371/journal.pbio.1001292)
Supplement: Table S11 — Spearman rank correlations among Total, Resident, and Endemic richness for different taxa across 110 km quadrants (N = 9,253). For richness definitions, see Table S2. (DOC) [file pbio.1001292.s015.doc]

**Table S11: Spearman rank correlations among *Total*, *Resident* and *Endemic* richness for different taxa across 110km quadrats (N= 9,253).** For richness definitions see Table S2.
