# Supplementary material for: Global Gradients in Vertebrate Diversity Predicted by Historical Area-Productivity Dynamics and Contemporary Environment
Source: PLoS Biol. 2012 Mar 27;10(3):e1001292. doi: 10.1371/journal.pbio.1001292 (PMC3313913; doi:10.1371/journal.pbio.1001292)
Supplement: Table S12 — Prediction success (r 2) of bioregion-level models of Total and Resident richness of 110 km grid cell assemblages (N = 9,253) based on general linear models. Grid cell richness is first fitted by the predictions for Resident species richness (“[Bioregion] Predicted richness,” see Table 1), and then additionally by Area of the bioregion, and grid-cell-level relative productivity (CellPropProductivity, calculated as proportion of maximum grid cell productivity in the region). Pseudo-r 2 values of observed versus fitted are listed. (DOC) [file pbio.1001292.s016.doc]

**Table S12: Prediction success (**r^2^**) of bioregion level models of *Total* and *Resident* richness of 110km grid cell assemblages (N = 9,253) based on general linear models**. Grid cell richness is first fitted by the predictions for *Resident* species richness (‘[Bioregion] Predicted richness’, see Table 1), and then additionally by *Area* of the bioregion, and grid-cell level relative productivity (*CellPropProductivity*, calculated as proportion of maximum grid cell productivity in the region). Pseudo-r^2^ values of observed vs. fitted are listed.
