# Supplementary material for: Global Gradients in Vertebrate Diversity Predicted by Historical Area-Productivity Dynamics and Contemporary Environment
Source: PLoS Biol. 2012 Mar 27;10(3):e1001292. doi: 10.1371/journal.pbio.1001292 (PMC3313913; doi:10.1371/journal.pbio.1001292)
Supplement: Table S13 — Prediction success of bioregion-level models of Total and Resident richness of 110 km grid cell assemblages (N = 9,253) based on mixed effects models. Grid cell richness is first fitted by the predictions for Resident species richness (“[Bioregion] Predicted richness,” see Table 1) and then additionally by bioregion Area, and grid-cell-level relative productivity (CellPropProductivity). Bioregion is fitted as a random effect, and the slope and strength of “[Bioregion] Predicted richness” and “[Bioregion] Predicted richness+Area” as fixed effects are assessed. Pseudo-r 2 values of observed versus fitted are listed. The additional effect of grid cell productivity was evaluated by fitting it as additional fixed effect with a globally constant slope (CellPropProductivity) and by allowing the relationship with richness to vary within regions as a random slope (CellPropProductivity var). (DOC) [file pbio.1001292.s017.doc]

**Table S13: Prediction success of bioregion level models of Total and Resident richness of 110km grid cell assemblages (N = 9,253) based on mixed effects models.** Grid cell richness is first fitted by the predictions for *Resident* species richness (‘[Bioregion] Predicted richness’, see Table 1), and then additionally by bioregion *Area*, and grid-cell level relative productivity (*CellPropProductivity*). Bioregion is fitted as a random effect, and the slope and strength of ‘[Bioregion] Predicted richness’ and ‘[Bioregion] Predicted richness + *Area*’ as fixed effects are assessed. Pseudo-r^2^ values of observed vs. fitted are listed. The additional effect of grid cell productivity was evaluated by fitting it as additional fixed effect with a globally constant slope (*CellPropProductivity*) and by allowing the relationship with richness to vary within regions as a random slope (*CellPropProductivity*_var_).
